# Bactericidal and Synergistic Effects of *Lippia origanoides* Essential Oil and Its Main Constituents against Multidrug-Resistant Strains of *Acinetobacter baumannii*

**DOI:** 10.1021/acsomega.4c07565

**Published:** 2024-10-16

**Authors:** Alisson T. da Silva, Ana Elisa C. M. Cândido, Edilson do C. M. Júnior, Gutiele N. do É, Marigilson P. S. Moura, Renata de F. S. Souza, Milena L. Guimarães, Rodolfo de M. Peixoto, Helinando P. de Oliveira, Mateus M. da Costa

**Affiliations:** † Animal Microbiology and Immunology Laboratory, Universidade Federal do Vale do São Francisco (UNIVASF), Campus Agricultural Sciences, Petrolina, Pernambuco 56300-000, Brazil; ‡ College of Pharmaceutical Sciences (CFARM), Universidade Federal do Vale do São Francisco (UNIVASF), Av. José de Sá Maniçoba, Centro, Petrolina, Pernambuco 56304-205, Brazil; § Laboratory of Impedance Spectroscopy and Organic Materials, Institute of Materials Science, Universidade Federal do Vale do São Francisco (UNIVASF), Juazeiro, Bahia 48902-300, Brazil

## Abstract

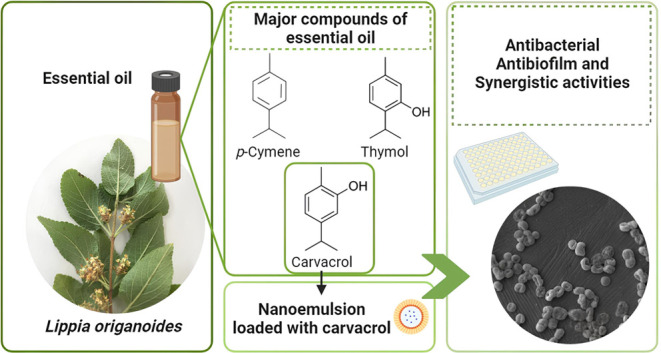

Bacterial resistance in *Acinetobacter baumannii* is a significant public health challenge, as these bacteria can evade multiple antibiotics, leading to difficult-to-treat infections with high mortality rates. As part of the search for alternatives, essential oils from medicinal plants have shown promising antibacterial potential due to their diverse chemical constituents. This study evaluated the antibacterial, antibiofilm, and synergistic activities of the essential oil of *Lippia origanoides* (EOLo) and its main constituents against multidrug-resistant clinical isolates of *A. baumannii*. Additionally, the antibacterial and antibiofilm potential of a nanoemulsion containing carvacrol (NE-CAR) was assessed. EOLo was extracted through hydrodistillation, and its components were identified via gas chromatography coupled with mass spectrometry. The *A. baumannii* isolates (*n* = 9) were identified and tested for antimicrobial susceptibility using standard disk diffusion methods. Antibacterial activity was determined by broth microdilution, while antibiofilm activity was measured using colorimetric methods with crystal violet and scanning electron microscopy. Synergism tests with antibiotics (meropenem, ciprofloxacin, gentamicin, and ampicillin+sulbactam) were performed using the checkerboard method. The primary constituents of EOLo included carvacrol (48.44%), *p*-cymene (14.58%), and thymol (10.16%). EOLo, carvacrol, and thymol demonstrated significant antibacterial activity, with carvacrol showing the strongest effect. They were also effective in reducing biofilm formation, as was NE-CAR. The combinations with antibiotics revealed significant synergistic effects, lowering the minimum inhibitory concentration of the tested antibiotics. Therefore, this study confirms the notable antibacterial activity of the essential oil of *L. origanoides* and its constituents, especially carvacrol, suggesting its potential as a therapeutic alternative for *A. baumannii* infections.

## Introduction

Bacterial infections are a leading cause of disease worldwide, exacerbated by the alarming rise in bacterial resistance in recent years.^1^ The species *Acinetobacter baumannii* represents a significant threat due to its genetic variability and diverse virulence factors, including adherence, persistence in hospital environments, and biofilm formation.^2,3^ As an opportunistic pathogen, *A. baumannii* can occasionally colonize human skin without causing disease,^4^ but it becomes pathogenic in Intensive Care Unit (ICU) patients, particularly those with burns, trauma, or under mechanical ventilation.^5,6^ Furthermore, carbapenem-resistant strains have been identified as a critical priority for the development of new antibiotics by the World Health Organization (WHO).^7^

Biofilm formation is a key factor contributing to increased bacterial resistance to antibiotics. These microbial communities, protected by an extracellular matrix, provide bacteria with a defensive environment that significantly enhances their resistance to antibiotics.^8^ In addition to facilitating survival against antimicrobial treatments, biofilms also promote the horizontal transfer of resistance genes among the bacteria presente.^9^ Thus, strategies focused on developing new antimicrobials and preventing biofilm formation are crucial.^10^

A promising approach against resistant bacteria is the use of phytotherapeutics, particularly essential oils extracted from medicinal plants.^11^ These oils are volatile liquids obtained from various plant parts and contain a variety of chemical compounds, such as terpenes and phenylpropanoids, known for their antibacterial properties.^12^ Combining essential oils with antibiotics has proven effective against resistant pathogens, broadening the antibacterial spectrum and reducing the need for high doses, thereby decreasing toxicity.^13,14^ In this context, nanoemulsions have emerged as a promising strategy to enhance the potential of essential oils.^15^ These nanoemulsions can increase the stability, solubility, and bioavailability of active compounds, boosting their antibacterial effects and facilitating clinical use.^16^

*Lippia origanoides*, also known as alecrim-pimenta, has attracted interest due to its antibacterial properties.^17^ The essential oils extracted from this plant, rich in compounds such as carvacrol and thymol, have shown efficacy against a wide range of pathogenic bacteria, including *Escherichia coli*, *Campylobacter jejuni*, *Salmonella enterica* subsp. *enterica*, and *Staphylococcus aureus*(18) with notable antibiofilm effects highlighted in recent studies.^19,20^ This research aimed to evaluate the antibacterial, antibiofilm, and synergistic potential of the essential oil from *L. origanoides* leaves and its main constituents against multidrug-resistant human clinical isolates of *A. baumannii*. Additionally, the antibacterial and antibiofilm activities of a nanoemulsion containing carvacrol were investigated.

## Results and Discussion

### Yield and Chemical Composition of the Essential Oil

The yield of essential oil extracted from the leaves of *Lippia origanoides* was 11.73% (v/w), which is notably high compared to other aromatic plant species. In studies on essential oil extraction, yields typically range between 0.1 and 5% (v/w), depending on the plant species, cultivation conditions, and extraction methods employed.^21^ Therefore, the yield obtained in this study suggests significant potential for the commercial and industrial applications of this plant.

**Figure 1:**
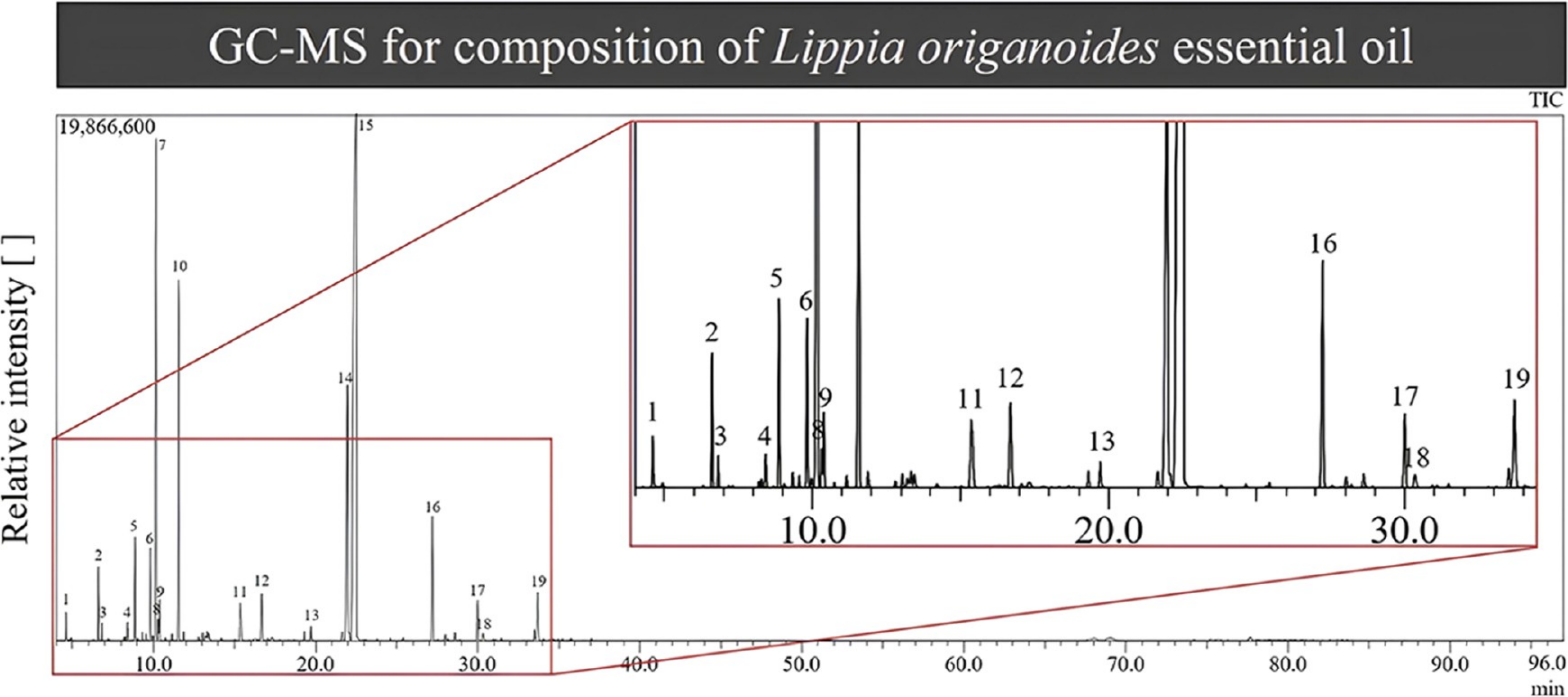
GC–MS chromatogram of *Lippia origanoides* essential oil.

Based on the GC-MS data (Figure 1), the compounds listed in Table 1 were identified. A total of 19 compounds were detected, accounting for 98.88% of the oil’s composition. The compound present in the highest proportion was carvacrol, followed by *p*-cymene, thymol, and γ-terpinene. Smaller amounts of β-caryophyllene, β-myrcene, 4-carene, and caryophyllene oxide were also detected.

**Table 1:**
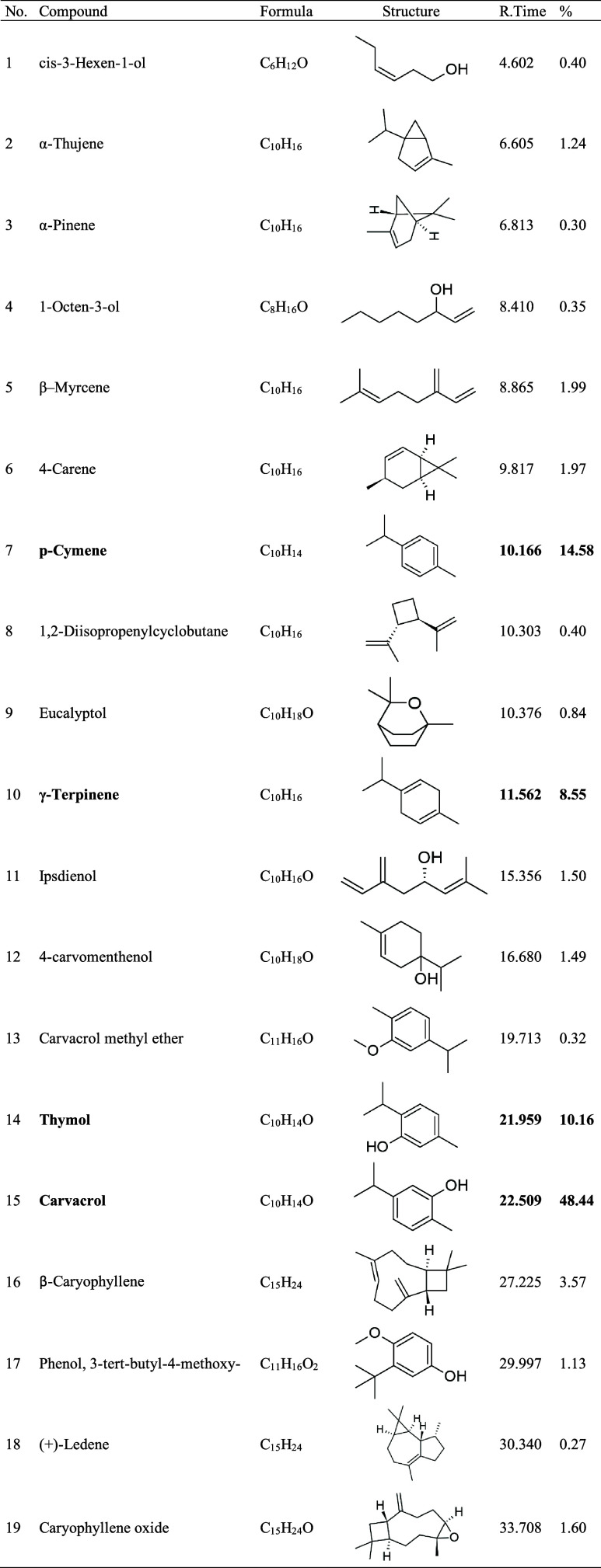
Chemical Composition of the Essential Oil Obtained from the Leaves of *Lippia origanoides* (EOLo)^a^

a R.Time: retention time; %: percentage.

The analysis of the chemical profile of the essential oil *L. origanoides* (EOLO) in this study revealed notable similarities with previous research. Oliveira et al.^22^ investigated the aerial parts of the plant in the state of Pará and identified carvacrol (38.6%), thymol (18.5%), *p*-cymene (10.3%), and γ-terpinene (4.1%) as the main components. Similar results were observed by Sarrazin et al.,^23^ who collected *Lippia origanoides* in the same region of Pará, reporting carvacrol as the major compound (47.2%), followed by thymol (12.8%), *p*-cymene (9.7%), and *p*-methoxythymol (7.4%). Additionally, Betancur-Galvis et al.^24^ evaluated essential oils from *L. origanoides* from different regions of Colombia, identifying a similar composition in two oils, with carvacrol (46.2%), *p*-cymene (12%), thymol (9.5%), and γ-terpinene (9.5%) being prominent. Thus, the predominance of carvacrol as the major component is noteworthy, reinforcing previous findings.

The data from this research corroborate the study conducted by Furlani et al.,^25^ who used samples from the same source (IF-SERTÃO) as the present study, showing a similar composition, with carvacrol (44.5%), *p*-cymene (14.06%), and γ-terpinene (12.43%). Several studies have reported the presence of chemotypes in *L. origanoides*, classified mainly into three categories based on the major component: chemotype A (*p*-cymene), chemotype B (carvacrol), and chemotype C (thymol).^23,26−28^

The distinct chemotypes may be attributed to genetic, environmental, and climatic factors, as well as the developmental stage of the plant.^29,30^ In this context, it is suggested that the EOLo examined in this study belongs to chemotype B, given the significant predominance of carvacrol (48.44%). These observations highlight the influence of multifactorial variables on the chemical composition of the EOLo, which may have implications for the efficacy and therapeutic properties of the essential oil. Additionally, the uniformity of chemotypes could be a crucial factor when considering the application of EOLo in different contexts, such as alternative therapies and pharmaceutical products. For example, EOLo chemotypes with high levels of carvacrol and thymol demonstrate more significant antibacterial activities, indicating that these compounds are crucial for antibacterial efficacy.^23,31^

### Characterization of the Nanoemulsion Containing Carvacrol

The preparation of nanoemulsions containing carvacrol (NE-CAR) aimed to explore their potential as an antimicrobial formulation. Nanoemulsions enhance the solubility of lipophilic compounds like carvacrol, improving bioavailability and antimicrobial efficacy.^15^ Additionally, they offer better stability by protecting the active compound from oxidative degradation, allow controlled release to extend antimicrobial action and reduce toxicity, and serve as a promising platform for future applications in treating infections.^16^ Based on the formulations previously described,^32^ NE-CAR was developed using the spontaneous emulsification method. Figure S1 (Supporting Information) shows the visual characteristics of the emulsions obtained. The nanoemulsion containing carvacrol (NE-CAR, A) exhibited a homogeneous appearance, with a milky and opaque texture, maintaining these stable properties for more than 60 days. In contrast, the control emulsion (NE-Control, B), without the addition of carvacrol, displayed similar characteristics but with a slight transparency in its appearance. These results indicate the feasibility of preparing the NEs and highlight the stability of the formulations over time.

Table 2 details the physical characteristics of the NEs. The average droplet size of NE-CAR and the control NE was 196.2 and 153.6 nm, respectively. The polydispersity index (PDI) for NE-CAR was 0.084, while the control NE showed a PDI of 0.176. The zeta potential of NE-CAR was −15.3 mV, while the control NE recorded a value of −10.9 mV.

**Table 2:** Physical Characteristics of the Nanoemulsions^a^

NE	particle size (d. nm)	PDI	ζ potential (mV)
NE + CAR	196.2	0.084	–15.3
NE (control)	153.6	0.176	–10.9

a NE: nanoemulsion; (d. nm): hydrodynamic diameter; PDI: polydispersity index; ζ: zeta; CAR: carvacrol; mV: millivolt.

In the present study, an NE-CAR formulation was introduced to evaluate its antibacterial and antibiofilm potential. The nanoemulsions produced had droplet sizes smaller than 200 nm. Droplet size in nanoemulsions directly affects stability, transparency, encapsulation of bioactive agents, antioxidant distribution, and antimicrobial efficacy.^33^ Smaller droplets tend to increase the stability and transparency of emulsions, improve encapsulation efficiency, and maintain good oxidative stability, although larger droplets may be more effective in antimicrobial activity due to the higher concentration of active agents at the oil–water interfaces.^34^ The nanoemulsion exhibited a satisfactory polydispersity index (PDI), with values below 0.2. This result suggests that the distribution of nanodroplets in the formulation is uniform and homogeneous, contributing to the overall stability of the nanoemulsion.^35^ The ζ potential represents the difference in electric charge between the surface of the droplets and the surrounding medium.^35^ These values indicate the stability of the suspensions, as values close to zero suggest a greater attraction between nanoparticles, which can lead to the formation of aggregates and reduced stability.^36^ These physical characteristics are crucial to ensuring the efficacy and practical applicability of the nanoemulsion.

### Antibacterial Activity

The results of the antibacterial activity tests are presented in Table 3. The EOLo showed MIC values ranging from 128 to 256 μg/mL and an MBC of 256 μg/mL. Thymol exhibited MIC values ranging from 64 to 128 μg/mL and MBC values from 64 to 256 μg/mL. Carvacrol demonstrated MIC values ranging from 32 to 64 μg/mL and MBC values from 64 to 128 μg/mL. *p*-Cymene did not exhibit any antibacterial effect at any of the tested concentrations.

**Table 3:** MIC and MBC of *Lippia origanoides* Essential Oil, Constituents, and Carvacrol Nanoemulsion on *A. baumannii* Isolates^a^

	EOLo		thymol		*p*-cymene		carvacrol		NE-CAR	
*A. baumannii* ID	MIC	MBC	MIC	MBC	MIC	MBC	MIC	MBC	MIC	MBC
199	128	256	64	64	>2048	>2048	32	64	256	256
280	256	256	128	128	>2048	>2048	64	64	256	256
285	256	256	128	128	>2048	>2048	64	64	256	256
288	256	256	128	128	>2048	>2048	64	64	256	256
301	256	256	128	128	>2048	>2048	64	128	256	256
307	128	256	128	128	>2048	>2048	64	64	256	256
309	256	256	128	256	>2048	>2048	64	64	256	256
314	256	256	128	256	>2048	>2048	64	128	256	256
324	256	256	128	128	>2048	>2048	64	64	256	256

a MIC: Minimum Inhibitory Concentration; MBC: Minimum Bactericidal Concentration; EOLo: *Lippia origanoides* Essential Oil; NE-CAR: Carvacrol Nanoemulsion.

When analyzing the average MIC and MBC values individually, it is noteworthy that carvacrol showed superior activity, with an average MIC of 60.4 ± 10.06 μg/mL and an MBC of 78.2 ± 26.73 μg/mL. Given this superior performance, carvacrol was selected for inclusion in the nanoemulsion formulation. The nanoemulsion containing carvacrol, although active, exhibited higher MIC and MBC values compared to free carvacrol, with both values at 256 μg/mL. The NE-control did not show bacteriostatic or bactericidal effects at the tested concentrations.

The remarkable antibacterial activity of the EOLo is largely attributed to the presence of oxygenated monoterpenes, particularly thymol and carvacrol.^22,37^ In this study, the antibacterial potential of these compounds was investigated separately. *p*-Cymene, which constitutes 14.58% of the EOLo, did not demonstrate bacteriostatic or bactericidal effects at the tested concentrations, suggesting that its role in the oil may be more of an adjuvant rather than a primary contributor to the observed antimicrobial activity. However, other studies have indicated that *p*-cymene possesses various pharmacological properties, including antioxidant, anti-inflammatory, antiparasitic, antidiabetic, antiviral, antitumoral, and analgesic effects.^38^

In contrast, both carvacrol and thymol exhibited strong bactericidal effects against multidrug-resistant *A. baumannii* strains. Additionally, carvacrol and thymol showed greater efficacy compared to the EOLo, suggesting that the presence of multiple components in the oil may sometimes lead to antagonistic effects, thereby reducing its overall antibacterial potential.^39^ Carvacrol is also the main component of the essential oil of *Thymus satureioides*, which was tested for its antibacterial activity against nine multidrug-resistant *A. baumannii* strains.^40^ The oil showed low minimum inhibitory and bactericidal concentrations, ranging from 780 to 3,125 μg/mL, and demonstrated high efficacy in biofilm inhibition and eradication.^40^ Cirino et al.^41^ also investigated the antibacterial effects of thymol and carvacrol on multidrug-resistant *A. baumannii* strains, revealing MIC values of 32–64 and 32–128 μg/mL, respectively, highlighting the immense antibacterial potential of these monoterpenes.

The mechanism of action of these two compounds is well-documented.^18,42^ Due to their hydrophobic characteristics, thymol and carvacrol can insert themselves into the fatty acid chains of cell membranes, disrupting membrane integrity by increasing permeability. This leads to conformational changes in the lipid bilayer, resulting in ruptures and leakage of cellular contents.^43,44^ Additionally, the hydroxyl group and the presence of double bonds in both thymol and carvacrol can participate in proton exchange, reducing the bacterial cytoplasmic membrane gradient. This results in the collapse of the proton motive force and ATP depletion, ultimately leading to cell death.^18,45^ A recent study also reported that thymol and carvacrol can downregulate genes related to ribosomal proteins and polypeptide chain processing.^41^ However, other intracellular targets may also be implicated in the antibacterial activity of these compounds, warranting additional research to fully elucidate their mechanisms.

Although this study did not include cytotoxicity tests on mammalian cells, previous research provides information into the safety profile of the main constituents of EOLo, such as carvacrol and thymol.^46,47^ These compounds have been widely studied and are used in formulations for human use, including food preservatives and pharmaceutical products.^48,49^ However, it is important to note that the toxicity of these molecules can vary significantly depending on their concentrations.^50^ For instance, while low to moderate concentrations are generally considered safe and exhibit therapeutic potential, higher concentrations have been associated with increased cytotoxicity in mammalian cells.^51^ Therefore, we recognize the need for future studies to rigorously assess cytotoxicity at various concentrations to ensure the safety of the oil, particularly at the higher doses required for its antibacterial activity.

### Quantification and Antibiofilm Activity

**Figure 2:**
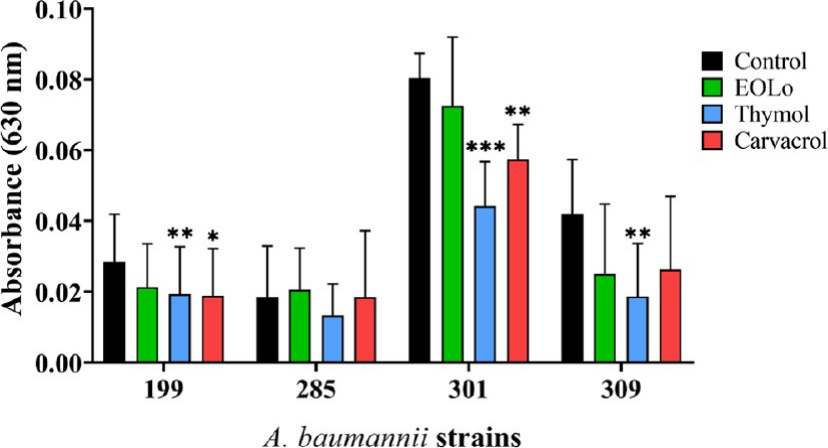
Biofilm production and interference of *L. origanoides* essential oil (EOLo) and its constituents (thymol and carvacrol) on the biofilm formation of *A. baumannii*. (*): *p* < 0,05; (**): *p* < 0,01; (***): *p* < 0,001.

All *A. baumannii* isolates evaluated demonstrated the ability to form biofilms on surfaces. Isolates 199, 285, and 309 were classified as weak producers, while isolate 301 was considered a moderate producer (Figure 2). The hydrophobicity and roughness of the surfaces, which are physicochemical characteristics, also play an important role in biofilm formation.^52^ Similarly, environmental factors, including but not limited to temperature, pH, other nutritional conditions, and the availability of a carbon source, such as lactose, also affect the ability of *A. baumannii* to form biofilms.^53^

The results obtained here corroborate studies indicating that biofilm formation by *A. baumannii* is greater in environmental isolates than in clinical isolates.^54,55^ Moreover, some studies report that a greater capacity to form biofilms may be related to higher levels of resistance in *A. baumannii*.^56^ However, Qi et al.^57^ reported that non-MDR isolates exhibited more robust biofilm formation, indicating that isolates with higher resistance levels (MDR) tend to form weaker biofilms, similar to what was observed in our study. Another study also did not identify a statistically significant relationship between biofilm production in MDR and non-MDR *A. baumannii* isolates.^58^ Thus, biofilm likely acts as a defense mechanism, primarily in bacteria more susceptible to the action of antibiotics, especially in hospital environments.^57^

Regarding antibiofilm activity (Figure 2), thymol showed a statistically significant reduction (*p* < 0.01) in biofilm formation for three isolates (199, 301, and 309), with a decrease ranging from 32.2 to 55.5%. Carvacrol also exhibited inhibitory effects, reducing biofilm formation in isolates 199 and 301 by 28.5% and 33.7%, respectively. On the other hand, the EOLo did not show a significant impact on *A. baumannii* biofilm formation.

Other studies have also reported that carvacrol and thymol are capable of reducing biofilm formation in various bacterial species.^59^ A recent study evaluated different subinhibitory concentrations of oregano essential oil (*Lippia Graveolens*), carvacrol, and thymol on biofilm formation in *A. baumannii*, revealing that carvacrol reduced biofilm formation by up to 95%, while thymol also showed a significant reduction.^60^ In another study, carvacrol and thymol demonstrated antibiofilm activity against colistin heteroresistant clinical isolates of *A. baumannii*.^61^ The mechanisms by which these molecules reduce biofilm formation may be related to the inhibition of twitching motility, reduced production of quorum sensing signals, inhibition of the initial adhesion of bacteria to surfaces, and reduced expression of genes related to the production of pili and extracellular matrix proteins.^59,62,63^

**Figure 3:**
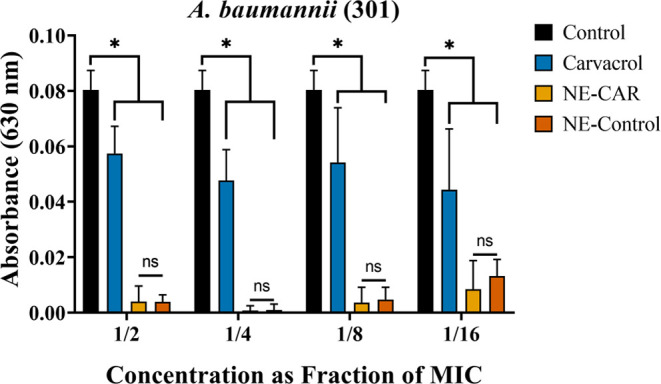
Interference of nanoemulsion with carvacrol (NE-CAR) and free carvacrol on biofilm formation in *A. baumannii* strain 301. *Statistically significant difference (*p* < 0.05); ns = not significant.

Regarding the activity of the nanoemulsion containing carvacrol compared to carvacrol in its free form (Figure 3), the nanoemulsion showed promising effects at all tested concentrations (1/2, 1/4, 1/8, and 1/16 MIC), significantly reducing biofilm formation in strain 301. This strain was selected for this analysis due to the notable reduction in biofilm formation caused by free-form carvacrol. However, no statistical difference was observed between NE-CAR and the control NE (without carvacrol), suggesting that the observed effects may be associated with the components used in the formulation. Additional results for strain 199, which exhibited similar trends, are provided in the Supporting Information (Figure S2) to avoid redundancy.

The use of nanoemulsions has proven to be a promising strategy for increasing the efficacy of antibiofilm compounds.^64^ Nanoemulsions are colloidal systems that allow the encapsulation of bioactive compounds at the nanoscale, significantly improving their solubility, stability, and bioavailability.^65^ Recent studies demonstrate that nanoemulsions containing carvacrol are more effective than the free compound against a variety of bacterial biofilms, including *Salmonella enteritidis*, *E. coli*, *S. aureus*, and *Listeria monocytogenes*.^66^ The mechanisms by which nanoemulsions enhance antibiofilm effects include sustained and controlled release of the active compound, allowing for prolonged and more effective action; efficient penetration into the deep layers of the biofilm due to the reduced particle size; and the ability to disrupt bacterial cell communication (quorum sensing), which is essential for the formation and maintenance of the biofilm.^67−69^ Therefore, the incorporation of antibiofilm compounds into nanoemulsions represents a significant advancement in the prevention and control of bacterial biofilms, offering a more efficient and targeted approach.

**Figure 4:**
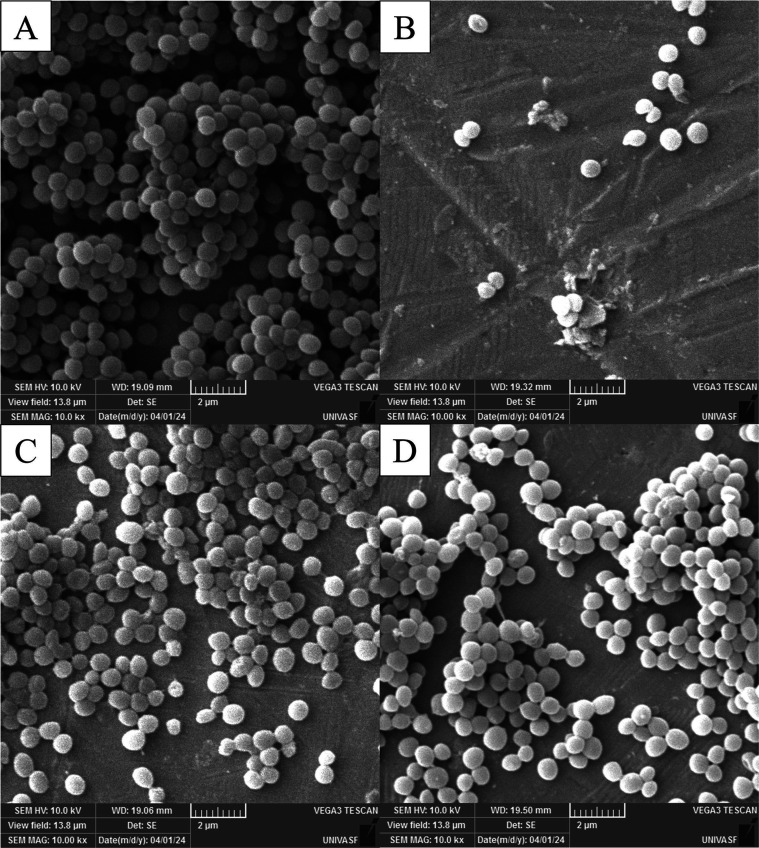
Scanning electron microscopy (SEM) analysis of *A. baumannii* (isolate 301) biofilm after different treatments. (A) Untreated cells (control); (B) Cells treated with EOLo; (C) Cells treated with carvacrol; (D) Cells treated with thymol. The scale bar represents 2 μm.

To observe the biological activity of these substances, analysis by Scanning Electron Microscopy (SEM) was performed. In Figure 4A, we observe the control condition of *A. baumannii* (301), in which cocco-bacillus-shaped cells are organized into three-dimensional communities, along with the secretion of the extracellular polymeric substance (EPS) matrix characteristic of biofilm formation. Isolate 301 was chosen because it was characterized as the highest biofilm producer among the isolates evaluated. Figure 4B–D show the cellular conditions and biofilm formation in the presence of the substances under test: EOLo, carvacrol, and thymol, respectively, at subinhibitory concentrations of 1/2 MIC. From the images, it is evident that there was a reduction in the number of bacterial cells, with severe morphological alterations, especially with carvacrol and thymol treatments (Figure 4C–D). These qualitative observations indicate that the substances under test led to a change in biofilm formation status from “moderate producer” to “weak producer.″ The differences observed between the quantitative and visual results suggest that while thymol and carvacrol are effective in reducing biofilm, EOLo may have limitations in its action due to its complex composition and possible interactions between its components. SEM evaluation can also reveal reductions in cell density and biofilm structure that are not detectable by traditional quantitative methods, such as the crystal violet method.

### Antibiotic Susceptibility Profile and Synergistic Activity

All *A. baumannii* clinical isolates selected for this study were characterized as multidrug-resistant (MDR), with resistance to more than three classes of antibiotics, as shown in Table S1 (Supporting Information). The *A. baumannii* strain 199 exhibited the highest multidrug-resistant index (MAR) of 0.875, being resistant to seven out of eight tested antibiotics. To quantitatively evaluate resistance to Meropenem, ciprofloxacin, gentamicin, and ampicillin/sulbactam, the broth microdilution method was employed (Table S2, Supporting Information). All *A. baumannii* strains exhibited resistance to Meropenem (breakpoint ≥8 μg/mL) and ciprofloxacin (breakpoint ≥4 μg/mL), with MIC values ranging from 64 to 256 and 32 to 128 μg/mL, respectively. The *A. baumannii* strain 199 was the only one resistant to gentamicin (breakpoint ≥16 μg/mL), with an MIC value of 16,384 μg/mL, and resistant to ampicillin/sulbactam (breakpoint ≥32/16 μg/mL), with values of 32/16 μg/mL.

The synergistic interactions between EOLo and its main constituents (thymol and carvacrol) with the antibiotics Meropenem, ciprofloxacin, and gentamicin against multidrug-resistant *A. baumannii* strains showed significant decreases in MIC values of these antibiotics. This is summarized in Table 4, where EOLo and thymol reduced, on average, 75, 81.25, and 75% of the MIC of Meropenem, ciprofloxacin, and gentamicin, respectively, while carvacrol did this, on average, by 71.87, 78.12, and 75%, respectively. These results pointed out that all three natural compounds had intense synergistic actions with these antibiotics. In turn, the combination with ampicillin+sulbactam showed an indifferent effect, indicating no significant enhancement of the antibiotic’s action. These findings reinforce these compounds’ potential as adjunct therapy in treating infections caused by resistant *A. baumannii*. More detailed information, including strain-specific responses, can be found in the Supporting Information (Tables S3–S5).

**Table 4:** Summary of Synergistic Effects between EOLo, Thymol, Carvacrol, and Selected Antibiotics against Multidrug-Resistant *A. baumannii*^a^

substance	antibiotics involved	average MIC reduction (%)
EOLo	Meropenem	75
	Ciprofloxacin	81.25
	Gentamicin	75
thymol	Meropenem	75
	Ciprofloxacin	81.25
	Gentamicin	75
carvacrol	Meropenem	71.87
	Ciprofloxacin	78.12
	Gentamicin	75

a MIC: Minimum inhibitory concentration; EOLo: essential oil *Lippia origanoides*.

The increasing prevalence of multidrug-resistant *A. baumannii* strains is a major concern,^70^ with natural resistance to several antibiotics, including aminopenicillins and cephalosporins.^71^ For sensitive strains, carbapenems like Meropenem and imipenem are typically used;^72^ however, carbapenem-resistant strains have emerged globally, leading to the use of more toxic alternatives like colistin and polymyxin B.^73,74^ Our study underscores the alarming antimicrobial resistance in strains from Petrolina (Pernambuco, Brazil), revealing limited treatment options.^75^ Understanding regional resistance patterns, influenced by local demographic and healthcare factors, is crucial for infection control and rational antibiotic use.^76,77^ Moreover, local strains should be used in developing new antimicrobials, as they reflect specific environmental and epidemiological conditions, ensuring treatment efficacy tailored to the region.^78^

Combination therapy is frequently used in the treatment of infections caused by *A. baumannii*, aiming to broaden the spectrum of antimicrobial activity and restore the efficacy of drugs previously considered obsolete.^14^ The combination of different antimicrobials, whether natural or synthetic, allows for a coordinated attack on various cellular targets or different growth stages, hindering bacterial defense against this multifaceted assault.^79^ In this study, the synergistic effect observed in the combination of antimicrobials (Meropenem, ciprofloxacin, and gentamicin) with the natural compounds EOLo, thymol, and carvacrol against *A. baumannii* is noteworthy. A significant reduction in the antibiotics’ MIC, ranging from 75 to 87.5%, was observed when these antibiotics were combined with the natural substances. Therefore, research aimed at increasing bacterial sensitivity and addressing the global challenge of bacterial resistance is indispensable.^14^ Furthermore, the results of this study align with several other studies, which have demonstrated that EOLo, carvacrol, and thymol are valuable compounds in combination therapy, enhancing the efficacy of various antibiotics.^80−83^

The global priority of finding new antibacterial agents is underscored by the prevalence of multidrug-resistant *A. baumannii* strains. In this study, the essential oil of *L. origanoides* and its main constituents, thymol and carvacrol, demonstrated remarkable antibacterial activities against *A. baumannii* strains. Additionally, carvacrol and thymol interfered with biofilm formation by *A. baumannii*. Furthermore, the formulation of a nanoemulsion containing carvacrol proved to be an effective vehicle, enhancing its stability while maintaining its bactericidal and antibiofilm efficacy. The essential oil of *L. origanoides*, thymol, and carvacrol exhibited significant synergistic effects when combined with Meropenem, ciprofloxacin, and gentamicin, highlighting their potential as therapeutic adjuvants. Given these results, carvacrol shows the most promise for further testing, particularly due to its superior efficacy both in its free form and when formulated in a nanoemulsion, as well as its significant impact on biofilm formation. These findings provide strong guidance for the antimicrobial application of these molecules, potentially serving as a foundation for developing more effective treatments against multidrug-resistant *A. baumannii* strains.

## Experimental Section

### General Experimental Procedures

For the maintenance of bacterial cultures and antimicrobial tests, the following media and reagents were used: Brain Heart Infusion (BHI) broth, BHI agar, Mueller Hinton (MH) broth, MH agar, and Trypticase Soy Broth (TSB), all sourced from Kasvi (São José dos Pinhais, PR, Brazil). The 2,3,5-triphenyltetrazolium chloride (TTC) was obtained from ACS Científica (Sumaré, SP, Brazil), while dimethyl sulfoxide (DMSO) was acquired from Neon (Suzano, SP, Brazil). Carvacrol (natural, 99%, d = 0.977 g/mL at 25 °C), thymol (98.5%, *d* = 0.965 g/mL at 25 °C), and p-cymene (99%, *d* = 0.86 g/mL at 25 °C) were purchased from Sigma-Aldrich (St. Louis, MO). The sodium salts of the antibiotics Meropenem, ciprofloxacin, and gentamicin were also sourced from Sigma-Aldrich. Ampicillin+sulbactam was obtained from Aurobindo Pharma (Hyderabad, Telangana, India).

Preparation of Nanoemulsions: The following materials were used for the preparation of nanoemulsions: sorbitan monooleate and POE 20 sorbitan monooleate, both obtained from Sigma-Aldrich. The medium-chain triglyceride (MCT) oil (*d* = 0.94 g/mL at 20 °C) was kindly provided by Brasquim (Porto Alegre, RS, Brazil).

### Plant Material, Extraction, and Characterization of *Lippia origanoides* Essential Oil

Aerial parts of *L. origanoides* were collected on March 13, 2023, at the Organic Medicinal Garden of the Federal Institute of Sertão Pernambucano, under the coordinates 9°20′17.2″S 40°41′56.4″W, located at the Zona Rural Campus, Km 22, N4, Petrolina, PE, Brazil. Specimens were identified and deposited in the Vale do São Francisco Herbarium (HVASF) under accession number 24998. Additionally, access to the material was registered in SisGen (Registration No. A6F8E32).

In the Chemistry Laboratory (IF-SERTÃO), only the leaves were separated from the aerial parts, and the fresh plant material (451.57 g) was subjected to hydrodistillation in a Clevenger apparatus for 2 h at 80 °C to obtain the essential oil. After extraction, the essential oil was stored in an amber glass container, protected from light, and refrigerated at 8 °C. The essential oil yield was calculated using the following formula: Yield (%) = (volume obtained (mL)/fresh mass (g)) × 100.

The chemical profile of EOLo was analyzed using a gas chromatograph coupled to a mass spectrometer (GC-MS, Shimadzu, GCMS-QP2020). An Agilent Technologies DB-5MS column (30 m × 0.25 mm × 0.25 μm; Agilent, Santa Clara) was used, and the sample was diluted in dichloromethane (1 μL/mL). Helium was used as the carrier gas at a flow rate of 3.0 mL/min, with 1.0 μL of the sample injected (split ratio of 1:10). The column temperature was increased from 50 to 280 °C at a rate of 3 °C/min. To remove the diluent peak (dichloromethane), a cutoff time of 4.0 min was applied. The mass spectra (MS) were obtained by electron ionization (EI) at 0.84 kV of ionization energy, and the spectrometer was operated in SCAN mode, covering a mass range from 37 to 660 *m*/*z*. The constituents were identified by comparing the mass spectra and fragments with the spectral libraries Wiley9, NIST08, and FFNSC1.3 available in the equipment’s software.

### Preparation of the Nanoemulsion Containing Carvacrol

The nanoemulsion (NE) formulation was prepared using the low-energy spontaneous emulsification method as described by Lima et al.^32^ The organic phase, composed of carvacrol, MCT oil, and surfactants (POE 20 sorbitan monooleate and sorbitan monooleate), was initially weighed and placed under constant magnetic stirring. After complete homogenization, the organic phase was added dropwise to the aqueous phase (phosphate buffer solution, pH ∼ 7.4) at room temperature, with constant magnetic stirring. After emulsification, the nanoemulsions were stored in glass bottles and refrigerated (2–8 °C). In this study, the surfactant-to-organic phase weight ratio and the percentage of the aqueous phase were kept constant at 1:1 (w/w) and 80%, respectively, and the amount of carvacrol added was fixed at 3.0% w/v. A control nanoemulsion (NE-Control) without carvacrol was similarly prepared using the same parameters.

### Characterization of the Nanoemulsion Containing Carvacrol

The hydrodynamic diameter and polydispersity index (PDI) of the NE droplets were measured using photon correlation spectroscopy (PCS) at 25 °C and a scattering angle of 173° (Zetasizer Nano ZS, Malvern Instruments, Worcestershire, UK). All samples were diluted 1:100 in ultrapure water. The data obtained were analyzed using the Zetasizer software (v3.30, Malvern Instruments, Worcestershire, UK).

The zeta potential (ζ) of the NE was measured by electrophoretic mobility using a Zetasizer Nano ZS (Malvern Instruments, Worcestershire, UK). The Smoluchowski model was applied to estimate the ζ potential from electrophoretic mobility. Analyses were conducted at 25 °C, with samples diluted 1:100 in ultrapure water. The reported values are the mean ± SEM of three different batches of each colloidal dispersion.

### Bacterial Isolates

Nine isolates (*n* = 9, ID: 199, 280, 285, 288, 301, 307, 309, 314, 324) of *A. baumannii* were used, obtained from the bacterial library of the University Hospital of the Federal University of Vale do São Francisco (HU-UNIVASF), located in Petrolina-PE, Brazil. The clinical samples were collected from human patients by the HU-UNIVASF team between December 2017 and October 2018, with approval from the research ethics committee of the Federal Rural University of Pernambuco (UFRPE), under number 4,652. The isolates were also registered in SisGen under code A92B495. These strains were identified using the Phoenix automated system (Becton Dickinson, Franklin Lakes, NJ).

For the preparation of bacterial stocks, the isolates were plated on BHI agar, and a colony from each isolate was inoculated into 5 mL of BHI broth and incubated for 24 h at 37 °C. Afterward, 750 μL of each bacterial suspension was added to 750 μL of 80% glycerol (1:1 ratio) in cryotubes and stored at −20 °C.

### Antimicrobial Susceptibility

The antimicrobial susceptibility of five isolates was analyzed using the disk diffusion method on agar, following the M100 protocol.^84^ A panel of eight antimicrobials representing six different classes was tested: cefepime 30 μg and ceftazidime 30 μg (cephalosporins); ciprofloxacin 5 μg (fluoroquinolone); doxycycline 30 μg and tetracycline 30 μg (tetracyclines); gentamicin 10 μg (aminoglycoside); Meropenem 10 μg (carbapenem); and sulfamethoxazole/trimethoprim 25 μg (folate pathway antagonist). All antimicrobial discs were obtained from CECON (Centro de Controle e Produtos para Diagnóstico, São Paulo, SP, Brazil).

Inocula were prepared by adding 5 μL of the bacterial stock to 10 mL of MH broth, followed by incubation at 37 °C for 24 h. Then, the OD at 600 nm of each isolate was measured and adjusted to a concentration of 1.5 × 10^6^ CFU/mL. Each suspension was spread on the surface of the MH agar using a swab, and the antimicrobial discs were aseptically added. After 24 h of incubation at 37 °C, the plates were removed and photographed. The diameters of the inhibition zones were analyzed using ImageJ software. The susceptibility results (sensitive, intermediate, and resistant) followed the parameters defined by M100.^84^ Bacterial isolates that exhibited resistance to three or more classes of antimicrobials were considered multidrug-resistant (MDR).

The determination of the Multiple Antibiotic Resistance Index (MARI) was based on the protocol used by Ayandele et al.,^85^ where the number of antibiotics to which a bacterial isolate is resistant (*a*) is divided by the total number of antibiotics tested (*b*), using the following formula: MARI = *a*/*b*.

### Antibacterial Activity

Treatment solutions for both EOLo and individual compounds were meticulously prepared in 5% DMSO, resulting in a final concentration of 4.096 μg/mL. These solutions were stored at 8 °C and protected from light until testing. Additionally, a DMSO solution in water, maintaining the same proportion, was used as a control for the antibacterial activity of the diluent.

The evaluation of antibacterial activity was obtained through the determination of the minimum inhibitory concentration (MIC) and the minimum bactericidal concentration (MBC), following the broth microdilution method according to the M07-A9 protocol.^86^ The test involved the distribution of 100 μL of MH broth into 96-well microtitration plates (OLEN, São José dos Pinhais, Brazil). Then, 100 μL of the test substance stock solutions (EOLo, thymol, carvacrol, *p*-cymene, and NE-CAR) were added to the first well and, after homogenization, were transferred to the second well, and so on. Concentrations ranging from 2.048 to 16 μg/mL were obtained for all test substances, except NE-CAR, which ranged from 4.096 to 32 μg/mL. The same method was applied for antibiotics, with concentrations ranging from 1.024 to 0.25 μg/mL, except for isolate 199, where the initial concentration of gentamicin was 16.384 μg/mL due to its high resistance.

For inoculum preparation, 5 μL of the bacterial stock were inoculated into 10 mL of MH broth and incubated at 37 °C for 24 h. The optical density (OD) at 600 nm (K37–UV/vis, Kasvi) of each isolate was then measured and adjusted to a concentration of 1.5 × 10^6^ CFU/mL. From this suspension, 10 μL were inoculated into the wells of the microplates. The material was incubated at 37 °C for 24 h under aerobic conditions. Next, a 10 μL aliquot from each well was plated on the surface of MH agar. After 24 h of incubation at 37 °C, the MBC was defined as the lowest concentration capable of causing bacterial death. Then, 30 μL of TTC were added to each well, and the plate was incubated for 1 h at 37 °C. A color change of the wells to pink/red indicates the metabolic activity of the microorganisms; thus, the MIC was determined by verifying the lowest concentration capable of inhibiting bacterial growth. Each assay was performed in triplicate. Sterility and bacterial viability controls were included.

### Quantification of Biofilm Production

To assess whether bacterial isolates produce biofilm, the methodology of Merino et al.^87^ and Stepanović et al.^88^ was followed, with modifications. Five microliters of each bacterial stock were inoculated into 5 mL of MH broth and incubated at 37 °C for 24 h. OD was measured at 600 nm, and the *A. baumannii* isolates were adjusted in TSB to achieve a bacterial concentration of 6 × 10^6^ CFU/mL. Next, 195 μL of TSB was added to sterile flat-bottom 96-well microplates (TPP – Techno Plastic Products, Trasadingen, Switzerland), along with 5 μL of the bacterial suspension. The microplate was then incubated at 37 °C for 24 h. After this period, the microplate was washed three times with 200 μL of sterile distilled water and inverted on paper towels to dry for 5 min at room temperature. The biofilm was fixed by adding 150 μL of analytical grade methanol (ACS Científica) to each well for 20 min at room temperature. After this time, the microplate contents were discarded, and it was left to dry overnight in an inverted position on paper towels at room temperature. The wells were then stained with 200 μL of 0.1% crystal violet (Synth, Diadema, São Paulo, Brazil) for 5 min. The wells were then washed three times with 200 μL of distilled water. Finally, 200 μL of an alcohol-acetone mixture (80:20 v/v; ACS Científica) was added to each well, and absorbance was measured at 630 nm using a microplate reader (LMR-96, LOCCUS, Cotia, São Paulo, Brazil). TSB medium served as both negative and sterility control. The assays were performed in triplicate.

According to the OD values obtained, the isolates were classified following the criteria of Stepanović et al.^89^ as nonbiofilm producers (OD_S_ < OD_NC_), weak producers (OD_NC_ < OD_S_ < 2xOD_NC_), moderate producers (2xOD_NC_ < OD_S_ < 4xOD_NC_), or strong biofilm producers (OD_S_ > 4xOD_NC_), where OD_S_ represents the optical density of the sample and OD_NC_ represents the optical density of the negative control.

### Antibiofilm Activity

The protocol for biofilm inhibition was based on the methodologies of Merino et al.^87^ and Nostro et al.,^90^ with modifications. For inoculum preparation, 5 μL of the bacterial stock was added to 5 mL of MH broth, followed by incubation at 37 °C for 24 h. The OD at 600 nm of each isolate was then measured and adjusted in TSB to obtain a concentration of 3 × 10^5^ CFU/mL. Next, 100 μL of the bacterial suspension was added to a 96-well microplate (TPP), along with 100 μL of essential oil solutions or isolated compounds to achieve a final concentration of 1/2 MIC. Additionally, carvacrol and nanoemulsions (with/without carvacrol) were evaluated at different concentrations: 1/2, 1/4, 1/8, and 1/16 of the MIC. After 24 h of incubation at 37 °C, the microplates underwent the same washing, fixation, staining, and absorbance reading processes as in the biofilm quantification assay. Each assay was conducted in triplicate.

### Scanning Electron Microscopy

To evaluate the effect of the test substances (EOLo, carvacrol and thymol) on *A. baumannii* cells, Scanning Electron Microscopy (SEM) was used, following the protocol previously described.^91^ The 301 strain was selected for analysis.

First, a sterile stainless steel support was placed in the wells of a 24-well microplate (Kasvi) to promote bacterial adherence. Next, the inoculum was prepared by adding 5 μL of the bacterial stock to 5 mL of MH broth, followed by incubation at 37 °C for 24 h. The OD of each isolate was measured at 600 nm and adjusted to a concentration of 3 × 10^5^ CFU/mL in TSB broth. Then, 500 μL of the bacterial suspension, along with 500 μL of the test solutions, were added to the wells, resulting in a final subinhibitory concentration of 1/2 MIC. For the untreated control, 500 μL of the bacterial suspension with 500 μL of sterile TSB medium were added.

After incubation for 24 h at 37 °C, the metal structures were washed twice with saline solution (0.85%) for 1 min and then immersed in 1% glutaraldehyde (Êxodo Científica, Sumaré, São Paulo, Brazil) for 3 h. The stainless steel structures were then immersed in a series of increasing ethyl alcohol concentrations (50, 70, 80, 90, and 99.5%, ACS Científica), each for 20 min. Finally, the samples were immersed in analytical grade acetone (ACS Científica) for 5 min and allowed to air-dry at room temperature before microscopic analysis.

For microscopic analysis, the stainless steel support was mounted on 3.2 mm diameter stubs and coated with a thin layer of gold. The samples were then inserted into the VEGA3 SEM (Tescan, Brno, Czech Republic), and images were obtained at 10,000× magnification.

### Synergism by the Checkerboard Method

To assess the synergistic potential of EOLo and its major compounds (carvacrol and thymol) with the antibiotics Meropenem, ciprofloxacin, gentamicin, and ampicillin+sulbactam, the Checkerboard test was performed following the method previously described.^92^ Only isolates that were classified as resistant to the tested antibiotics were selected for evaluating synergistic effects.

A bacterial suspension was adjusted in MH broth to a concentration of 1.5 × 10^6^ CFU/mL. Then, 100 μL of MH broth was added to all wells. In column no. 1, 100 μL of the diluted antibiotic was added at a concentration of 4× the MIC value for serial dilution, transferring 100 μL horizontally to column 6. In row “A,″ 100 μL of the solution corresponding to the natural compounds was added at a concentration of 2× the MIC value and serially diluted vertically down to row F. The final concentrations obtained were 1×, 1/2×, 1/4×, 1/8×, 1/16×, and 1/32× of the MIC value for both substances. In each well of the microtiter plate, 10 μL of the bacterial suspension was added. In wells H7, H8, and H9, only bacteria and MH broth were added as bacterial viability controls. In wells H10, H11, and H12, only 100 μL of MH broth was added, serving as a sterility control for the process. The microplates were incubated for 24 h at 37 °C. The CTT indicator was then added, and the plate was incubated for approximately 1 h before the result was read.

The interpretation of the synergistic effect of each antimicrobial and their combinations was determined by calculating the fractional inhibitory concentration index (FIC), using the following formula: FIC = MIC of the product when tested alone/MIC of the combined product. The sum of the FICs was used to classify the effects of the substance association, according to Lee et al.^92^ synergistic action (FIC ≤ 0.5), additive (0.5 < FIC < 1), indifferent (1 < FIC < 2), and antagonistic (FIC ≥ 2).

### Statistical Analysis

All statistical analyses were performed using GraphPad Prism 7 software. The results were plotted as mean ± standard deviation (SD). Differences between groups were determined by analysis of variance (ANOVA) with Tukey’s post-test, using a *p*-value <0.05.

## Supporting Information Available

Visual aspects of nanoemulsions, their interference in A. baumannii (strain 199) biofilm formation, susceptibility profiles of isolates, and interactions of EOLo and its constituents with antibiotics (PDF)
