# Supplementary material for: Bactericidal and Synergistic Effects of Lippia origanoides Essential Oil and Its Main Constituents against Multidrug-Resistant Strains of Acinetobacter baumannii
Source: ACS Omega. 2024 Oct 16;9(43):43927–39. doi: 10.1021/acsomega.4c07565 (PMC11525495; doi:10.1021/acsomega.4c07565)
Supplement: Supplementary file 1 — ao4c07565_si_001.pdf [file ao4c07565_si_001.pdf]

SUPPORTING INFORMATION

Bactericidal and Synergistic Effects of *Lippia origanoides* Essential Oil and Its Main Constituents Against Multidrug-Resistant Strains of *Acinetobacter baumannii*

Alisson T. da Silva, [*, a] *Ana Elisa C. M. Cândido,* [a] *Edilson do C. M. Júnior,* [a] *Gutiele N. do É,* [a] *Marigilson P. S. Moura,* [b] *Renata de F. S. Souza,* [a] *Milena L. Guimarães,* [c] *Rodolfo de M. Peixoto,* [a] *Helinando P. de Oliveira* [c] *and Mateus M. da Costa* [*, a]

[a] Animal Microbiology and Immunology Laboratory, Universidade Federal do Vale do São Francisco (UNIVASF), Campus Agricultural Sciences, Petrolina, Pernambuco, 56300-000, Brazil.

[b] College of Pharmaceutical Sciences (CFARM), Universidade Federal do Vale do São Francisco (UNIVASF), Av. José de Sá Maniçoba, Centro, Petrolina, Pernambuco, 56304-205, Brazil.

[c] Laboratory of Impedance Spectroscopy and Organic Materials, Institute of Materials Science, Universidade Federal do Vale do São Francisco (UNIVASF), Juazeiro, Bahia, 48902-300, Brazil.

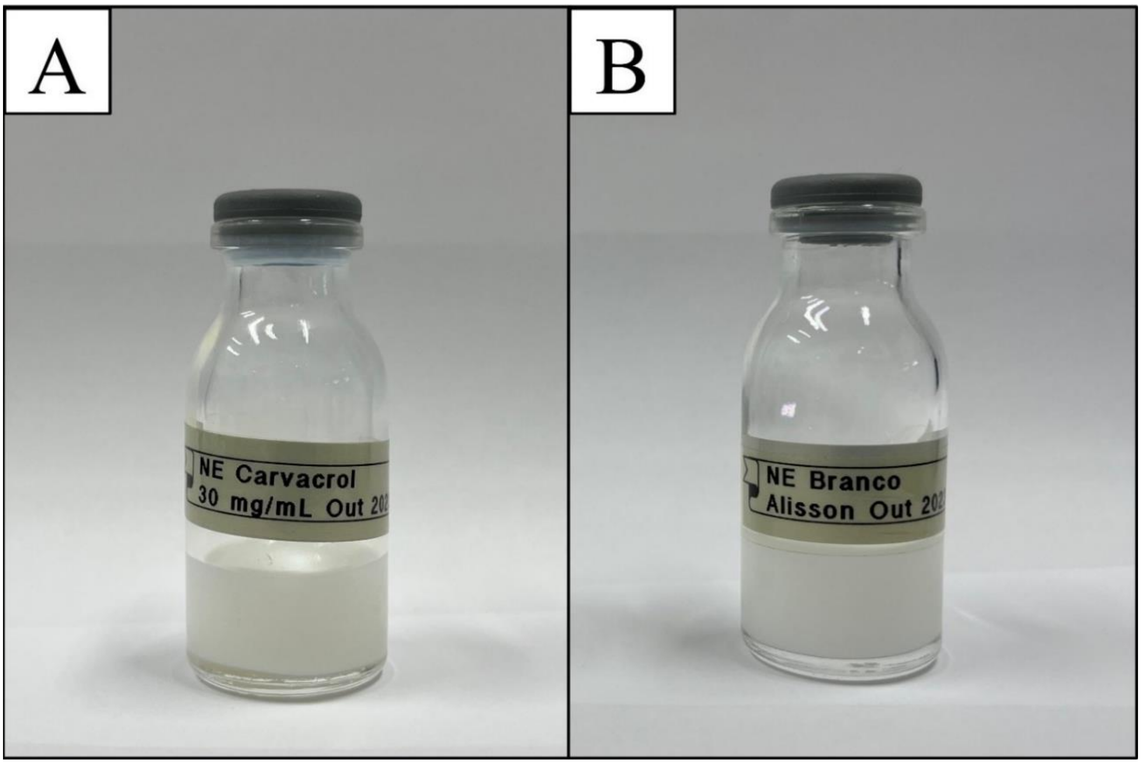

Figure S1. Visual aspects of the nanoemulsion containing carvacrol (A) and the control nanoemulsion (B) immediately after preparation.

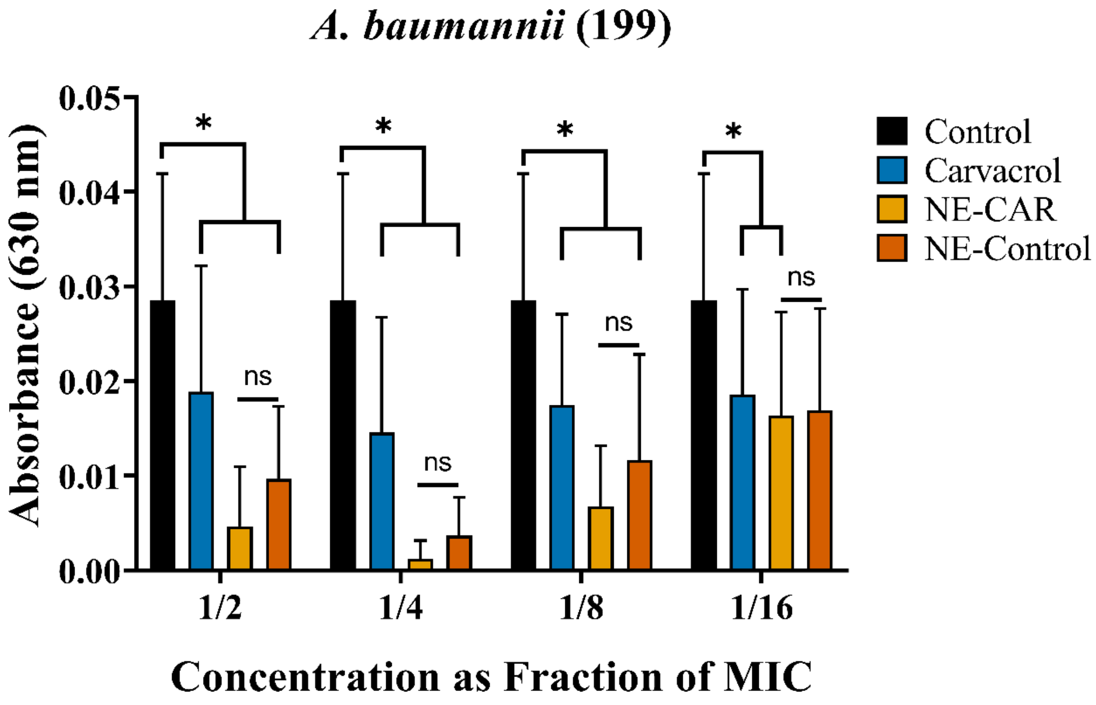

Figure S2. Interference of nanoemulsion with carvacrol (NE-CAR) and free carvacrol on biofilm formation in *Acinetobacter baumannii* strain 199. *Statistically significant difference ($p < 0.05$); ns = not significant.

Table S1. Antimicrobial susceptibility profile of *Acinetobacter baumannii* strains.

	A. baumannii				
ATB	199	285	301	309	324
CPM	0,0 ± 0,0 (R)	0,0 ± 0,0 (R)	0,0 ± 0,0 (R)	0,0 ± 0,0 (R)	11,3 ± 1,9 (R)
CAZ	0,0 ± 0,0 (R)	0,0 ± 0,0 (R)	0,0 ± 0,0 (R)	0,0 ± 0,0 (R)	0,0 ± 0,0 (R)
CIP	0,0 ± 0,0 (R)	0,0 ± 0,0 (R)	0,0 ± 0,0 (R)	0,0 ± 0,0 (R)	0,0 ± 0,0 (R)
DOX	9,7 ± 1.3 (R)	27,4 ± 0,9 (S)	25,7 ± 1,5 (S)	26,1 ± 1,2 (S)	24,3 ± 1,3 (S)
GEN	0,0 ± 0,0 (R)	11,9 ± 0,1 (R)	13,6 ± 1,0 (I)	13,8 ± 0,4 (I)	17,8 ± 1,0 (S)
MER	0,0 ± 0,0 (R)	0,0 ± 0,0 (R)	0,0 ± 0,0 (R)	0,0 ± 0,0 (R)	0,0 ± 0,0 (R)
SUT	0,0 ± 0,0 (R)	0,0 ± 0,0 (R)	0,0 ± 0,0 (R)	0,0 ± 0,0 (R)	18,5 ± 2,3 (S)
TET	16,3 ± 1,5 (S)	20,7 ± 1,8 (S)	18,4 ± 1,7 (S)	18,6 ± 1,6 (S)	16,0 ± 1,6 (S)
IRMA	0,875	0,75	0,625	0,625	0,5

Values presented as mean ± standard deviation in mm. ATB = Antibiotic; CPM = Cefepime; CAZ = Ceftazidime; CIP = Ciprofloxacin; DOX = Doxycycline; GEN = Gentamicin; MER = Meropenem; SUT = Sulfazotrim; TET = Tetracycline; SD = Standard deviation; MAR = Multiple antibiotic resistance index; (S) = sensitive; (I) = intermediate; (R) = resistant.

Table S2. Minimum inhibitory concentration (MIC) and minimum bactericidal concentration (MBC) of antimicrobials against clinical isolates of *Acinetobacter baumannii*.

A. baumannii ID	Antimicrobials (µg/mL)											
	Meropenem			Ciprofloxacin			Gentamicin			Ampicillin +Sulbactam		
	MIC	MBC	CLS.	MIC	MBC	CLS.	MIC	MBC	CLS.	MIC	MBC	CLS.
199	64	64	R	32	32	R	16,384	16,384	R	32/16	32/16	R
285	256	256	R	128	256	R	8	16	I	16/8	16/8	I
301	256	256	R	128	256	R	8	8	I	16/8	16/8	I
309	256	256	R	128	256	R	8	8	I	16/8	16/8	I
324	64	64	R	32	32	R	2	2	S	8/4	16/8	S

CLS = classification; (S) = sensitive; (I) = intermediate; (R) = resistant. Breakpoints for resistant: Meropenem ≥ 8 µg/mL; Ciprofloxacin ≥ 4 µg/mL; Gentamicin ≥ 16 µg/mL; Ampicillin+Sulbactam ≥ 32/16 µg/mL.

Table S3. Interaction of EOLo and its constituents (thymol and carvacrol) with meropenem against *Acinetobacter baumannii* isolates.

A. baumannii	Association	MIC (µg/mL)		∑IFI	Effect	MIC reduction %
		Individual	Combined			
199	Meropenem	64	16	0,312	Synergistic	75,0
	EOLo	128	8			93,8
	Meropenem	64	16	0,312	Synergistic	75,0
	Thymol	64	4			93,8
	Meropenem	64	16	0,312	Synergistic	75,0
	Carvacrol	32	2			93,8
285	Meropenem	256	64	0,312	Synergistic	75,0
	EOLo	256	16			93,8
	Meropenem	256	64	0,312	Synergistic	75,0
	Thymol	128	8			93,8
	Meropenem	256	64	0,312	Synergistic	75,0
	Carvacrol	64	4			93,8
309	Meropenem	256	64	0,312	Synergistic	75,0
	EOLo	256	16			93,8
	Meropenem	256	64	0,312	Synergistic	75,0
	Thymol	128	8			93,8
	Meropenem	256	128	0,562	Additive	50,0
	Carvacrol	64	4			93,8
324	Meropenem	64	16	0,312	Synergistic	75,0
	EOLo	256	16			93,8
	Meropenem	64	16	0,312	Synergistic	75,0
	Thymol	128	8			93,8
	Meropenem	64	8	0,25	Synergistic	87,5

Carvacrol	64	8	87,5

MIC = minimum inhibitory concentration; IFI: fractional inhibitory index; % reduction = (individual MIC / combined MIC) x 100/individual MIC; ΣIFI ≤ 0.5: synergism; ΣIFI > 0.5 and ≤ 1: additive; ΣIFI > 1 and ≤ 2: indifferent; ΣIFI > 2: antagonistic.

Table S4. Interaction of EOLo and its constituents (thymol and carvacrol) with ciprofloxacin against *Acinetobacter baumannii* isolates.

A. baumannii	Association	MIC (µg/mL)		∑IFI	Effect	MIC reduction %
		Individual	Combined			
199	Ciprofloxacin	32	8	0,312	Synergistic	75,0
	EOLo	128	8			93,8
	Ciprofloxacin	32	8	0,375	Synergistic	75,0
	Thymol	64	8			87,5
	Ciprofloxacin	32	8	0,375	Synergistic	75,0
	Carvacrol	32	4			87,5
285	Ciprofloxacin	128	16	0,187	Synergistic	87,5
	EOLo	256	16			93,8
	Ciprofloxacin	128	16	0,187	Synergistic	87,5
	Thymol	128	8			93,8
	Ciprofloxacin	128	16	0,25	Synergistic	87,5
	Carvacrol	64	8			87,5
309	Ciprofloxacin	128	16	0,25	Synergistic	87,5
	EOLo	256	32			87,5
	Ciprofloxacin	128	16	0,25	Synergistic	87,5
	Thymol	128	16			87,5
	Ciprofloxacin	128	32	0,312	Aditivo	75,0
	Carvacrol	64	4			93,8
324	Ciprofloxacin	32	8	0,312	Synergistic	75,0
	EOLo	256	16			93,8
	Ciprofloxacin	32	8	0,312	Synergistic	75,0
	Thymol	128	8			93,8
	Ciprofloxacin	32	8	0,312	Synergistic	75,0

Carvacrol	64	4	93,8

MIC = minimum inhibitory concentration; IFI: fractional inhibitory index; % reduction = (individual MIC / combined MIC) x 100/individual MIC; ΣIFI ≤ 0.5: synergism; ΣIFI > 0.5 and ≤ 1: additive; ΣIFI > 1 and ≤ 2: indifferent; ΣIFI > 2: antagonistic.

Table S5. Interaction of EOLo and its constituents (thymol and carvacrol) with gentamicin and ampicillin + sulbactam against *Acinetobacter baumannii* isolate.

A. baumannii	Association	MIC (µg/mL)		∑IFI	Effect	MIC reduction %
		Individual	Combined			
199	Gentamicin	16384	4096	0,5	Synergistic	75,0
	EOLo	128	32			75,0
	Gentamicin	16384	4096	0,375	Synergistic	75,0
	Thymol	64	8			87,5
	Gentamicin	16384	4096	0,375	Synergistic	75,0
	Carvacrol	32	4			87,5
	Amp + Sulbactam	32	32	1,062	Indifferent	0,0
	EOLo	128	8			93,8
	Amp + Sulbactam	32	32	1,062	Indifferent	0,0
	Thymol	64	4			93,8
	Amp + Sulbactam	32	32	1,125	Indifferent	0,0
	Carvacrol	32	4			87,5

MIC = minimum inhibitory concentration; IFI: fractional inhibitory index; % reduction = (individual MIC / combined MIC) x 100/individual MIC; ΣIFI ≤ 0.5: synergism; ΣIFI > 0.5 and ≤ 1: additive; ΣIFI > 1 and ≤ 2: indifferent; ΣIFI > 2: antagonistic.
